# Effects of Two Terpene Alcohols on the Antibacterial Activity and the Mode of Action of Farnesol against *Staphylococcus aureus*

**DOI:** 10.3390/molecules13123069

**Published:** 2008-12-09

**Authors:** Naoko Togashi, Yoshihiro Inoue, Hajime Hamashima, Akihito Takano

**Affiliations:** 1 Laboratory of Bio-Medicinal Pharmacology, Showa Pharmaceutical University, Machida, 194-8543 Tokyo, Japan; 2 Department of Microbiology, Showa Pharmaceutical University, Machida, 194-8543 Tokyo, Japan; 3 Laboratory of Kampo Medicine and Pharmacognosy, Showa Pharmaceutical University, Machida, 194-8543 Tokyo, Japan

**Keywords:** Terpene alcohol, Antibacterial activity, *Staphylococcus aureus*, Cell membrane.

## Abstract

We have studied changes in the antibacterial activity and the mode of action of farnesol against *Staphylococcus aureus* when two terpene alcohols with an aliphatic carbon chain were added, individually, to a bacterial suspension that contained farnesol. Geraniol increased the growth-inhibitory activity of farnesol, but suppressed its ability to damage cell membranes, which is one of the predominant features of the growth-inhibitory activity of farnesol. Geranylgeraniol decreased the growth-inhibitory activity of farnesol and also suppressed its cell-damaging activity. It is possible that the presence of a terpene alcohol can both enhance and suppress the antibacterial activity of farnesol, and even change its mode of action. Thus, it is important to study not only the antibacterial activity of each constituent of an essential oil but also the interactions between them in efforts to characterize the antibacterial activity of the essential oil.

## Introduction

Since resistance to antibiotics has become a serious problem, efforts have been made to identify novel compounds with antibacterial activity. Natural products, in particular, plant-derived compounds, have attracted considerable attention [1]. They are present in many traditional medicines and have been used for many years because of their apparent safety [1].

One of the most attractive essential oils, in this context, is tea tree oil (TTO), which is derived from *Melaleuca alternifolia* and has a broad spectrum of antibacterial and anti-inflammatory activities [2,3,4,5,6]. It has been suggested that the activity of TTO is due mostly to terpinen-4-ol, which is a major constituent [7,8,9]. However, Inoue *et al*. showed that myrcene, which is a minor constituent of TTO, might modify the activity of terpinen-4-ol and the activity of mixture of terpinen-4-ol and myrcene approaches that of TTO [10]. These observations suggested interactions among constituents of essential oils.

In this study, we investigated changes in the growth-inhibitory activity of farnesol against *S. aureus* FDA209P in the presence of another terpene alcohol with an aliphatic carbon chain. We showed previously that farnesol was the effective inhibitor of the growth of *Staphylococcus aureus* in studiesof the antibacterial activity of terpenes with an aliphatic carbon chain [11,12,13,14,15]. We used the “broth dilution with shaking” (BDS) method to estimate growth-inhibitory activity because of the low solubility of these compounds in water. We also measured leakage of K^+^ ions from bacterial cells since the mode of action of farnesol in growth inhibition includes damage to cell membranes.

## Results and Discussion

**Figure 1:**
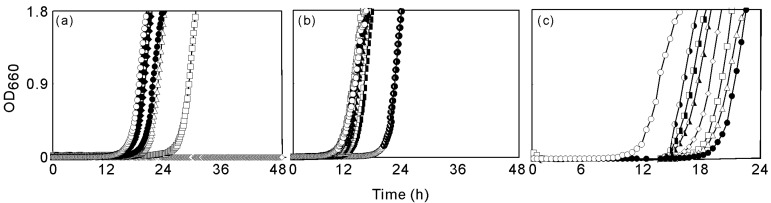
Effects of terpene alcohol on the growth of *S. aureus* FDA209P.

As we reported previously, farnesol exhibited antibacterial activity against *S. aureus* and the effects were dependent on the concentration of farnesol [15]. Farnesol suppressed the growth of *S. aureus* FDA209P for more than 48 h at concentrations of 10 μg mL^-1^ and higher (Figure 1a).

(a) farnesol, (b) geraniol and (c) geranylgeraniol

*S. aureus* FDA209P was inoculated into BHI broth medium that contained no terpene alcohol (○) or 0.16, 0.32, 0.64, 1.25, 2.5, 5, 10, 20, 40 or 80 (▲, ■, ◆, ●, △, □, ◇, ◮, ◨ or ◑) μg of terpene alcohol per mL. Concentrations of 0.16, 0.32 and 0.64 μg mL^-1^ were only used for farnesol.

Geraniol and geranylgeraniol also exhibited antibacterial activity against *S. aureus* FDA209P (Figure 1b and Figure 1c). However, neither terpene inhibited the growth of *S. aureus* FDA209P for 48 h. Thus, their activities were lower than that of farnesol. 

**Figure 2:**
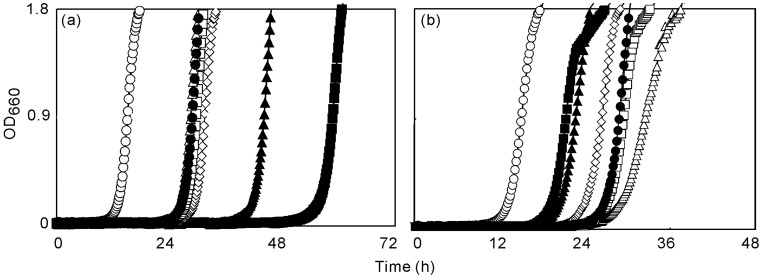
Effects of geraniol and geranylgeraniol on the growth inhibitory activity of farnesol to *S. aureus* FDA209P.

(a) geraniol-farnesol system, (b) geranylgeraniol-farnesol system. *S. aureus* FDA209P was inoculated into BHI broth medium that contained no farnesol (○) or 5 (●) μg of farnesol per mL with 5, 10, 20, 40 or 80 (△, □, ◇, ▲or ■) μg of geraniol or geranylgeraniol per mL.

Figure 2a shows the growth-inhibitory effects when geraniol was added to a bacterial suspension that contained farnesol at a fixed concentration of farnesol of 5 μg mL^-1^. To compare antibacterial activities, we calculated the delay in proliferation (DP) in each case, as listed in Table 1. DP was taken as the difference between the experimental and control cultures in terms of the time required to reach an OD_660_ of 0.9. Addition of geraniol increased the DP and the increase in DP was greater with higher concentrations of geraniol. When the concentration of geraniol was 80 μg mL^-1^, the growth of *S. aureus* FDA209P was inhibited for more than 48 h.

**Table 1:** Effect of geraniol and geranylgeraniol on the growth inhibitory and the membrane damaging activity of farnesol against *S. aureus* FDA209P.

Terpene	Ratio^a^	DP^b^ (h)	Leakage of K^+^ ions^c)^(x 10^-9^ mol μg^-1^)	Initial rate alcohol of leakage^c)^ (x 10^-10^ mol μg^-1 ^sec^-1^)
Farnesol + geraniol	1 : 0	13.8	267	8.8
	1 : 1	13.5	271	11.1
	1 : 2	14.5	395	13.2
	1 : 4	16.3	186	3.1
	1 : 8	29.0	173	2.4
	1 :16	43.7	N.T.^ d^	N.T.^ d^
Farnesol+geranylgeraniol	1 : 0	13.8	267	8.8
	1 : 1	17.2	28	1.1
	1 : 2	14.3	27	0.8
	1 : 4	11.3	33	0.8
	1 : 8	7.7	15	0.3
	1 :16	6.3	N.T.^d^	N.T.^d^

^a^ Concentration of farnesol was set at 5 and 10 μg mL^-1^ when the growth inhibitory and the leakage of K^+^ ions in response to mixed terpenes was observed, respectively; ^b^ delay in proliferation; ^c^ Assays were performed in triplicate. Results are shown as mean values; ^d^ not tested.

**Figure 3:**
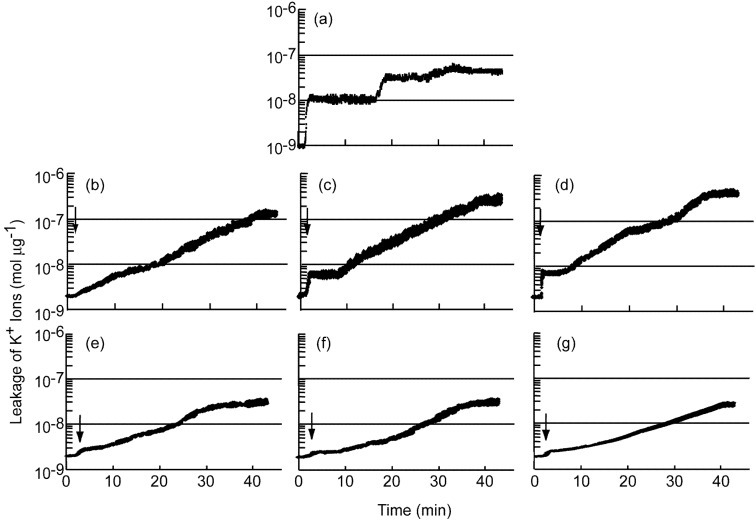
Changes in the concentration of K^+^ ions in a suspension of *S. aureus* FDA209P in response to mixture of farnesol and geraniol or geranylgeraniol.

Figure 2b shows the growth-inhibitory effects of geranylgeraniol in the presence of farnesol, at 5 μg mL^-1^, in a similar experiment. Addition of geranylgeraniol reduced the DP (Table 1), and the decrease in DP increased with increases in the concentration of geranylgeraniol added. Figure 3a shows changes in the concentration of K^+^ ions in a suspension of *S. aureus* FDA209P in response to addition of farnesol at 5 μg mL^-1^. The rapid increase in the concentration of K^+^ ions just after the addition of farnesol. We previously reported that the mode of action of antibacterial activity of farnesol was to damage bacterial cell membrane [16].

The final concentration of farnesol was 5 μg mL^-1^ (a), that of geraniol added was (b), 40; (c), 20; and (d), 10 μg mL^-1^ and that of geranylgeraniol was (e), 40; (f), 20; and (g), 10 μg mL^-1^.

Figure 3b, Figure 3c and Figure 3d show changes in the concentration of K^+^ ions in a suspension of *S. aureus* FDA209P in response to addition of a mixture of farnesol and geraniol. The concentration of farnesol was fixed at 5 μg mL^-1^ and the tested concentrations of geraniol were 10, 20 and 40 μg mL^-1^. The addition of geraniol affected the leakage of K^+^ ions. The rapid increase in the concentration of K^+^ ions just after the addition of the mixture of the terpene alcohols was suppressed with the extent of suppression related to the concentration of geraniol. The duration of the “plateau” also decreased. The amounts and initial rates of leakage of K^+^ ions are summarized at Table 1. When the concentration of geraniol was 10 μg mL^-1^, both the amount and the initial rate of leakage of K^+^ ions and, also the DP, increased slightly. When the concentration of geraniol was 20 μg mL^-1^ and higher, the initial rate decreased rapidly and the amount of K^+^ ions leaking from cells decreased gradually. In addition, the DP increased.

Figure 3e, Figure 3f and Figure 3g show changes in the concentration of K^+^ ions in a suspension of *S. aureus* FDA209P in a similar experiment with a mixture of farnesol and geranylgeraniol. Addition of the mixture of farnesol and geranylgeraniol increased the concentration of K^+^ ions in the bacterial suspension. The initial rate of leakage of K^+^ ions decreased with increases in the concentration of geranylgeraniol (Table 1). Then, the concentration of K^+^ ions in the bacterial suspension increased gradually. After approximately 40 min, the level of K^+^ ions reached a plateau.

In our examination of the interactions between terpene alcohols, we found that geranylgeraniol suppressed the antibacterial activity of farnesol and simultaneously moderated the damaging effects of farnesol on cell membranes (Table 1).

It was reported previously that geranylgeraniol has antibacterial activity against *S. aureus* FDA209P and that its mode of action involves damage to cell membranes, being similar to that of farnesol. The activity of geranylgeraniol depended on its concentration. However, at a certain concentration, the dependence on concentration of the antibacterial activity was reversed [17]. We concluded that geranylgeraniol has growth-accelerating activity that outweighs its ability to damage cell membranes when the concentration of geranylgeraniol is higher than the “turn-over” point. Therefore, we can explain the decrease in the antibacterial activity of farnesol upon the addition of geranylgeraniol, by postulating that the growth-accelerating activity of geranylgeraniol plays an important role and that geranylgeraniol is not a competitive inhibitor of farnesol. It is likely that a decrease in membrane-damaging activity led to the decrease in the antibacterial activity of farnesol against *S. aureus* FDA209P.

Geraniol plus farnesol increased the DP more than farnesol alone (Table 1). The growth-inhibitory effects of the mixture were not the sum of the growth-inhibitory effects of each constituent: the sum of the DP of the individual constituents was smaller than the DP of the mixture. Thus, geraniol promoted the antibacterial activity of farnesol.

The amount of K^+^ ions that leaked from cells did not increase with the increase in DP. In this context, the antibacterial activity of the mixture of farnesol and geraniol was bacteriostatic. This bacteriostatic effect did not induce the disruption of cell membranes at any time. The initial rate of leakage of K^+^ ions decreased when the concentration of geraniol was 20 or 40 μg mL^-1^. The tendency of the decrease in the initial rate of leakage of K^+^ ions was similar to that observed with geranylgeraniol plus farnesol. However, the DP increased with the addition of geraniol, even though the antibacterial activity did not involve damage to cell membranes. Some other mechanism might have enhanced the antibacterial activity of farnesol. Thus, the addition of geraniol might have changed the mode of action of the antibacterial activity of farnesol, enhancing its activity.

There are many reports of the antibacterial activities of plant-derived compounds, such as essential oils. To clarify the mode of action of such activities, each constituent is usually assayed separately. Our results reveal that terpenes, which are a major category of plant-derived compounds, might interact with each other and with bacterial cells to increase or decrease each other’s antibacterial activity. Thus, it is important to investigate not only single constituents but also combinations in studies of the antibacterial activities of plant-derived compounds. Further studies are needed to characterize the mechanisms of changes in antibacterial activities in systems that include mixtures of terpenes.

## Experimental

### Reagents and microorganism

*trans,trans*-Farnesol, geraniol and geranylgeraniol were purchased from Sigma Chemical Co. (St. Louis, MO, U.S.A.). *Staphylococcus aureus* FDA209P was used as the standard strain [11].

### “Broth dilution with shaking” (BDS) method

The BDS method was described previously [10] and applied as follows. The compound or mixture of compounds to be tested was added, at the indicated concentrations, to 10 mL aliquots of Brain Heart Infusion (BHI) broth (Difco, Detroit, MI) in L-tubes (17 mm i.d.; length of arms, 180 mm and 70 mm) without any solubilizing agent or surfactant. An aliquot of an overnight culture of *S. aureus* was added to each sample to give approximately 1 x 10^5^ colony-forming units (CFU) of *S. aureus* per mL. Each culture was incubated, with shaking at 40 rpm, in air for 24, 48 or 72 h at 37^o^C. The inhibitory activity of each tested compound or mixture was monitored turbidimetrically. The optical density at 660 nm (OD_660_) was determined with a biophotorecorder (TN-1520; Advantec, Tokyo, Japan). The delay in proliferation (DP) was calculated from a comparison with the growth curve generated from a control culture.

### Quantitation of leakage of K^+^ ions

The concentration of K^+^ ions in 4.0 mL of a suspension of cells (10^9^ CFU mL^-1^ initially) was determined with K^+^-selective and reference electrodes. A silver/silver chloride electrode was used as the reference electrode. The K^+^-selective electrode was prepared using valinomycin, as described by Katsu *et al*. [18], and the system for quantitation of K^+^ ions was constructed as reported previously [19]. Compounds were added to 100-μL aliquots of a suspension of cells. Each assay was performed at 37^o^C. Data were recorded digitally. Amounts and initial rates of leakage of K^+^ ions that leaked from cells were expressed in terms of bacterial mass. For estimations of the bacterial mass in each test suspension, bacterial cells were disrupted by sonication and centrifuged (30,000 rpm, 1 h, 4^o^C) and then the concentration of protein in the supernatant was determined as described by Bradford [20], with bovine serum albumin as the standard protein. The measurement was carried out at least three times. The results shown are typical of the results in each case.
